# High-throughput rapid-prototyping of low-cost paper-based microfluidics

**DOI:** 10.1038/s41598-017-02931-6

**Published:** 2017-06-15

**Authors:** Fariba Ghaderinezhad, Reza Amin, Mikail Temirel, Bekir Yenilmez, Adam Wentworth, Savas Tasoglu

**Affiliations:** 1 Department of Mechanical Engineering, University of Connecticut, Storrs, CT 06269 USA; 2 Department of Biomedical Engineering, University of Connecticut, Storrs, CT 06269 USA; 3 Department of Material Science and Engineering, University of Connecticut, Storrs, CT 06269 USA; 4 Institute of Materials Science (IMS), University of Connecticut, 97 North Eagleville Road, Storrs, CT 06269 USA; 5 The Connecticut Institute for the Brain and Cognitive Sciences, University of Connecticut, 337 Mansfield Rd, Storrs, CT 06269 USA; 6 Institute for Collaboration on Health, Intervention, and Policy (InCHIP), University of Connecticut, 2006 Hillside Road, Storrs, CT 06269 USA

## Abstract

Paper-based micro analytical devices offer significant advantages compared to the conventional microfluidic chips including cost-effectiveness, ease of fabrication, and ease of use while preserving critical features including strong capillary action and biological compatibility. In this work, we demonstrate an inexpensive, rapid method for high-throughput fabrication of paper-based microfluidics by patterning hydrophobic barriers using a desktop pen plotter integrated with a custom-made, low-cost paper feeder. We tested various types of commercial permanent markers and compared their water-resistant capabilities for creating hydrophobic barriers. Additionally, we studied the performance of markers with different types of paper, plotting speeds, and pattern dimensions. To verify the effectiveness of the presented fabrication method, colorimetric analysis was performed on the results of a glucose assay.

## Introduction

Utilizing paper as an easy-to-use and disposable platform for micro analytical devices has garnered considerable attention for point-of-care applications in clinical diagnostics^1, 2^, environmental protection and monitoring^3, 4^, food safety testing^5–7^, chemical education^8^, and forensic analysis. Some particularly attractive advantages of paper-based micro analytical devices are affordability, facile disposability, portability, and simple fabrication which facilitate mass-production^9–12^. Moreover, multiplexing these devices enables simultaneous execution of multiple assays on a single device without cross-contamination^13^. Sample transportation in paper-based microfluidics is controlled via patterned hydrophobic barriers and propagated by means of the strong capillary action of paper, obviating the need for pumps^9, 13, 14^. Several approaches including photolithography^6, 15^, laser etching^16, 17^, and plasma treatment^18^ have been extensively presented with well-characterized materials such as glass, silicon, and polymers for the fabrication of micro analytical devices. The limitations of these fabrication methods, including lengthiness and the need for expensive instruments and trained personnel, pose challenges regarding ease of mass fabrication and cost-effectiveness in both resource-limited regions and developed countries^11, 13, 19–21^.

Martinez *et al*. introduced the idea of using paper as a substrate with patterned hydrophobic barriers using a UV-polymerized photoresist^15^. Abe *et al*. developed a fabrication method based on inkjet printing for creating hydrophilic channels on hydrophobic paper^22^. Over the years, researchers have employed different hydrophobic agents including wax^23, 24^, permanent marker ink^11^, polydimethylsiloxane (PDMS)^25^, and alkylketene dimer (AKD)^18, 26^ for delimiting the barriers. Among these approaches, flexographic^14^ and inkjet printing^22, 27^ are promising for high-throughput and mass fabrication of paper-based microfluidics^28^ due to their advantages of affordability and simple single-step nature^11, 12^.

**Figure 1:**
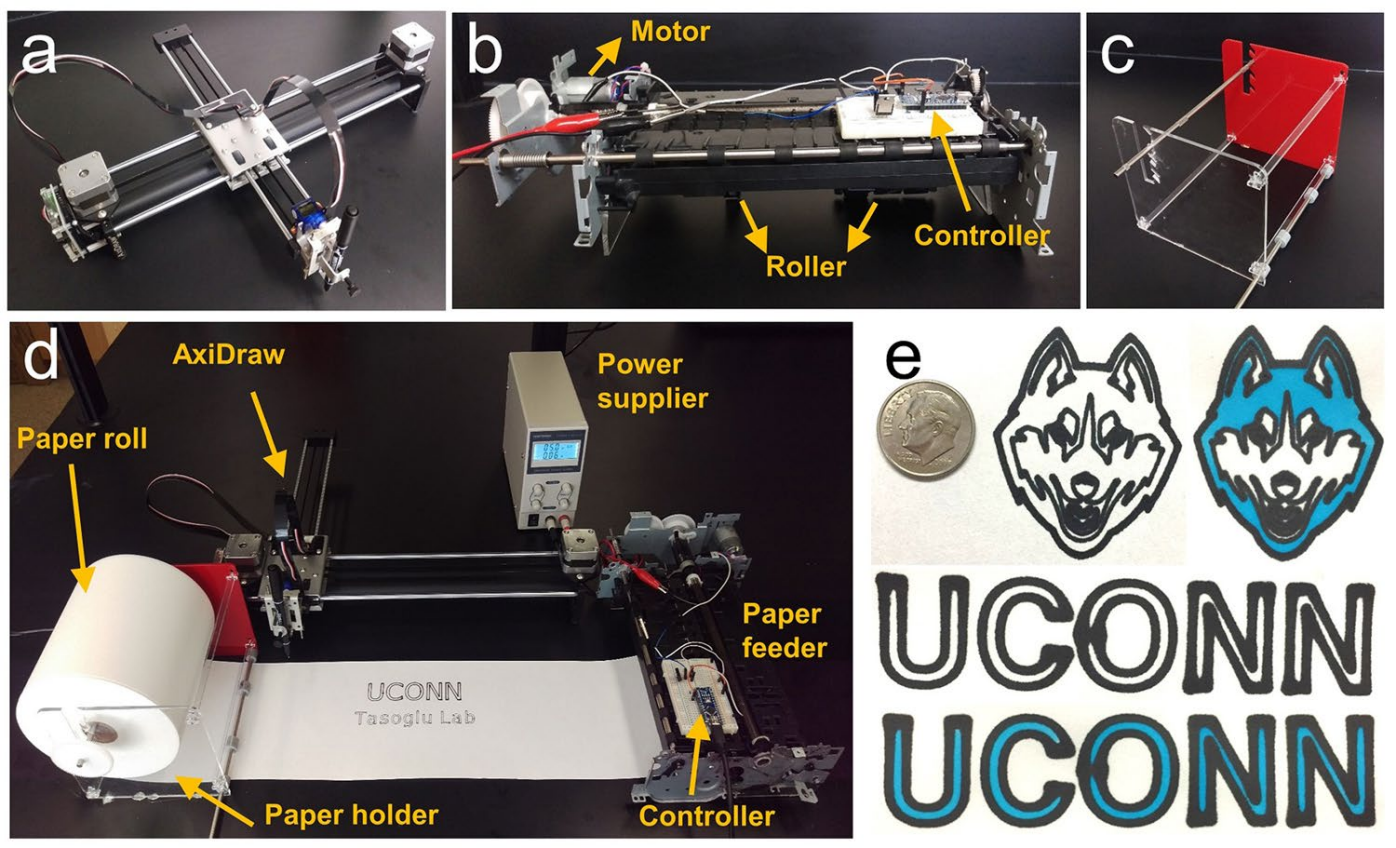
High-throughput rapid-prototyping of low-cost paper-based microfluidics. (**a**) Desktop pen plotter and (**b**) a customized paper feeder. (**c**) Holder of paper roll manufactured with a laser cutter. (**d**) Plotter integrated with the feeder. (**e**) Sample patterns plotted by desktop pen plotter and colored by liquid fabric dye.

In this work, we developed a pen-plotter-based approach, integrated with a custom-made, low-cost paper feeder (Fig. 1) for single-step patterning of hydrophobic barriers and high-throughput fabrication of paper-based microfluidics. We investigated the capacity of several commercial permanent markers to produce hydrophobic barriers on delicate task wipers and chromatography papers. To show the effectiveness of this fabrication method, we conducted a glucose assay and used colorimetric analysis to present the results. Despite the limitations of the colorimetric method, such as inhomogeneity of color distribution and background noise, this quantification method was employed owing to its easy operation, straightforward signal readout, and the quantifiable results^12, 27^. Since the intensity of the color in detection zones is a function of analyte concentration, quantification of results can easily be performed with a digital camera^12, 13^. Therefore, we used digital image processing to locate the detection zones and analyze the color intensities by using a MATLAB image processing script.

## Results

### Water-resistant capability of the markers

**Figure 2:**
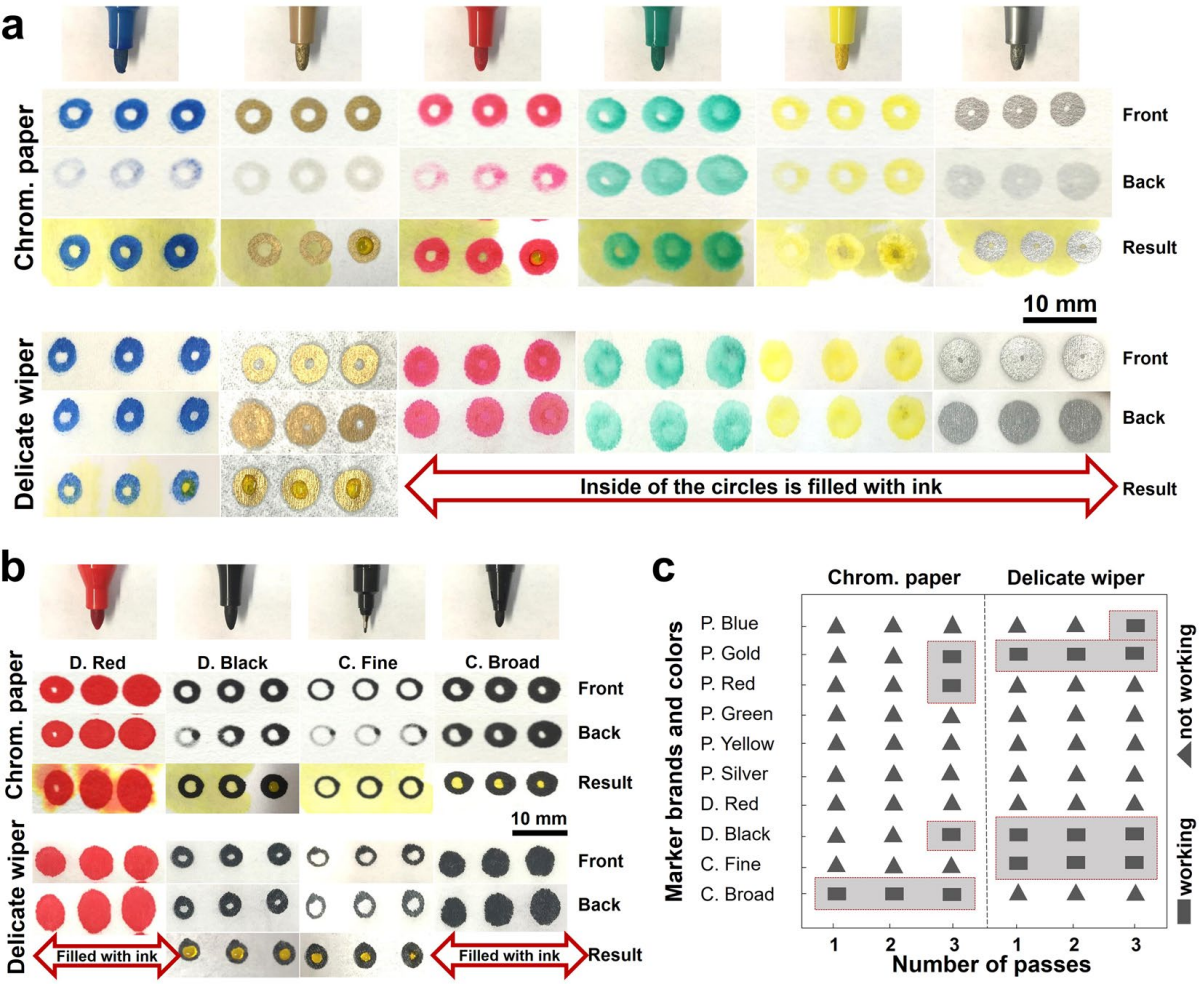
The water-resistant capability of markers. (**a**) Images of plotted patterns with Sipa markers with different colors on chromatography papers and delicate task wipers. From top to bottom, images were arranged to show the front and the back of the papers (with only inks), and results by spotting yellow aqueous food dye. (**b**) Images of plotted patterns with Deli and Comix markers on chromatography papers and delicate task wipers. (**c**) Performance chart for different markers and papers for single and multiple passes. Diameter of the circles is 4 mm.

**Figure 3:**
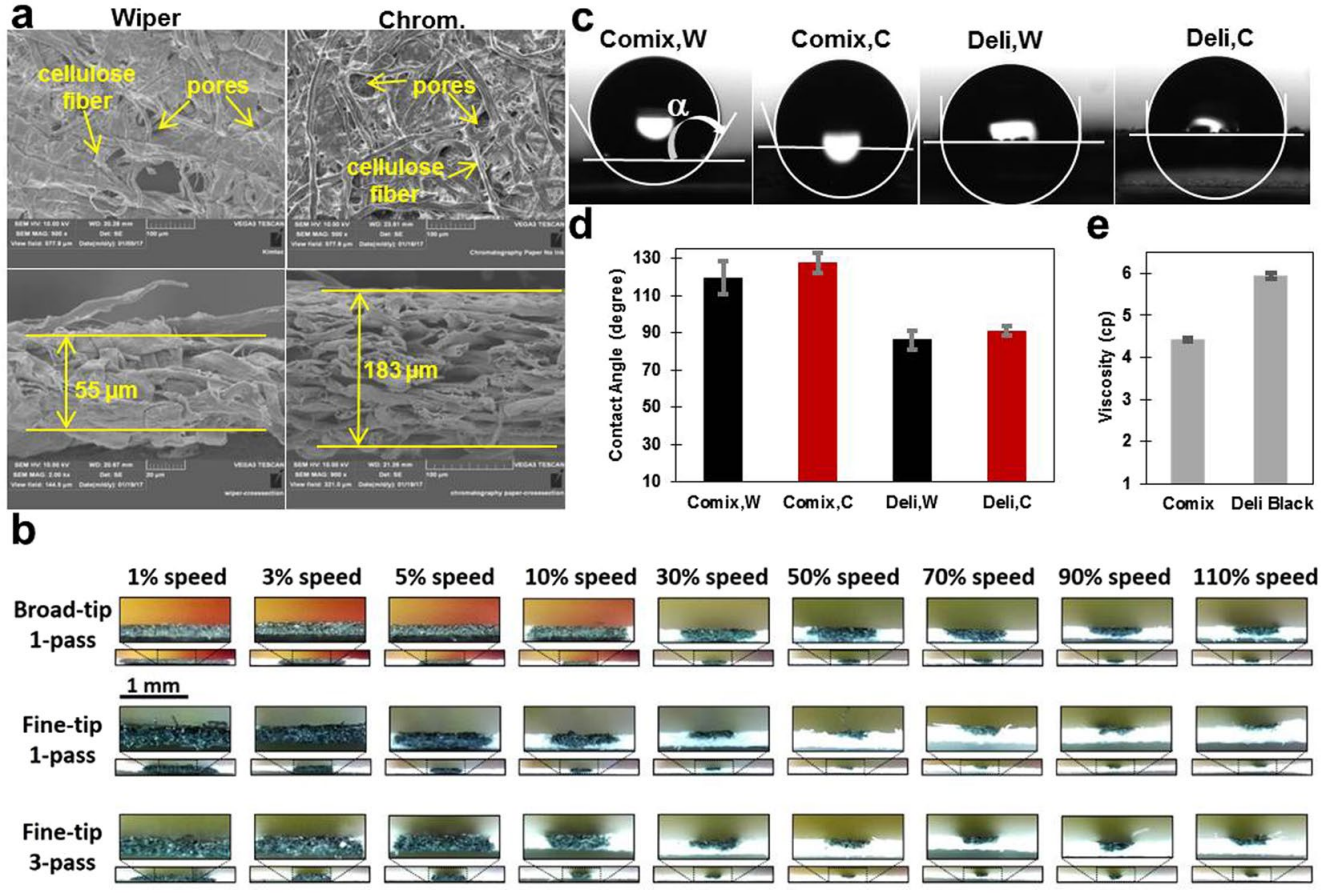
Characterization of paper substrates and inks. (**a**) SEM images of the surface and cross-section of delicate task wipers and chromatography paper. (**b**) Diffusing ink through chromatography paper at different plotting speeds from 1% (5.45 mm/s) to 110% (177 mm/s), and number of passes using fine and broad-tip of Comix marker. (**c**) Images of a 2-µl-droplet on different substrates covered with the inks. C and W stands for chromatography paper and Wipers, respectively. (**d**) Contact angle of a 2-µl-droplet on substrates covered with Comix and Deli ink (data are average of left and right contact angles). C and W stands for chromatography paper and Wipers, respectively. (**e**) Viscosity of Comix and Deli inks at 23 °C. Measurements are average of six repeats for both contact angle and viscosity.

We plotted patterns on both delicate task wipers and chromatography paper with all types of markers we used in this study (Fig. 2a,b). Majority of the patterns plotted on the front of the chromatography paper are not clearly visible from the back (Fig. 2a,b), showing that the amount of ink was not adequate to diffuse through the paper. However, the patterns on both the front and back of the delicate task wiper are almost identical since the wipers are thinner and have lower mesh density and ink can diffuse easer. This also explains why most of the circular patterns plotted on the wipers are filled with ink (Fig. 2a,b). The mesh density of both chromatography paper and wipers have been investigated using SEM imaging (Fig. 3a). The larger pore size and smaller thickness of wipers explain why inks penetrate better through delicate task wiper than chromatography paper.

The results of testing water-resistant capability of markers demonstrate that, only the patterns plotted with Comix broad-tip markers and 3 passes of Deli black markers are able to resist the aqueous solution on the chromatography paper (Fig. 2a,b). However, on the delicate task wiper, a single pass of Comix fine-tip or Deli black markers are able to contain aqueous solution of dye within the pattern. Figure 2c provides a summary of the performance of all markers used for plotting on two types of paper.

### The effect of the plotting speed and pattern dimensions

**Figure 4:**
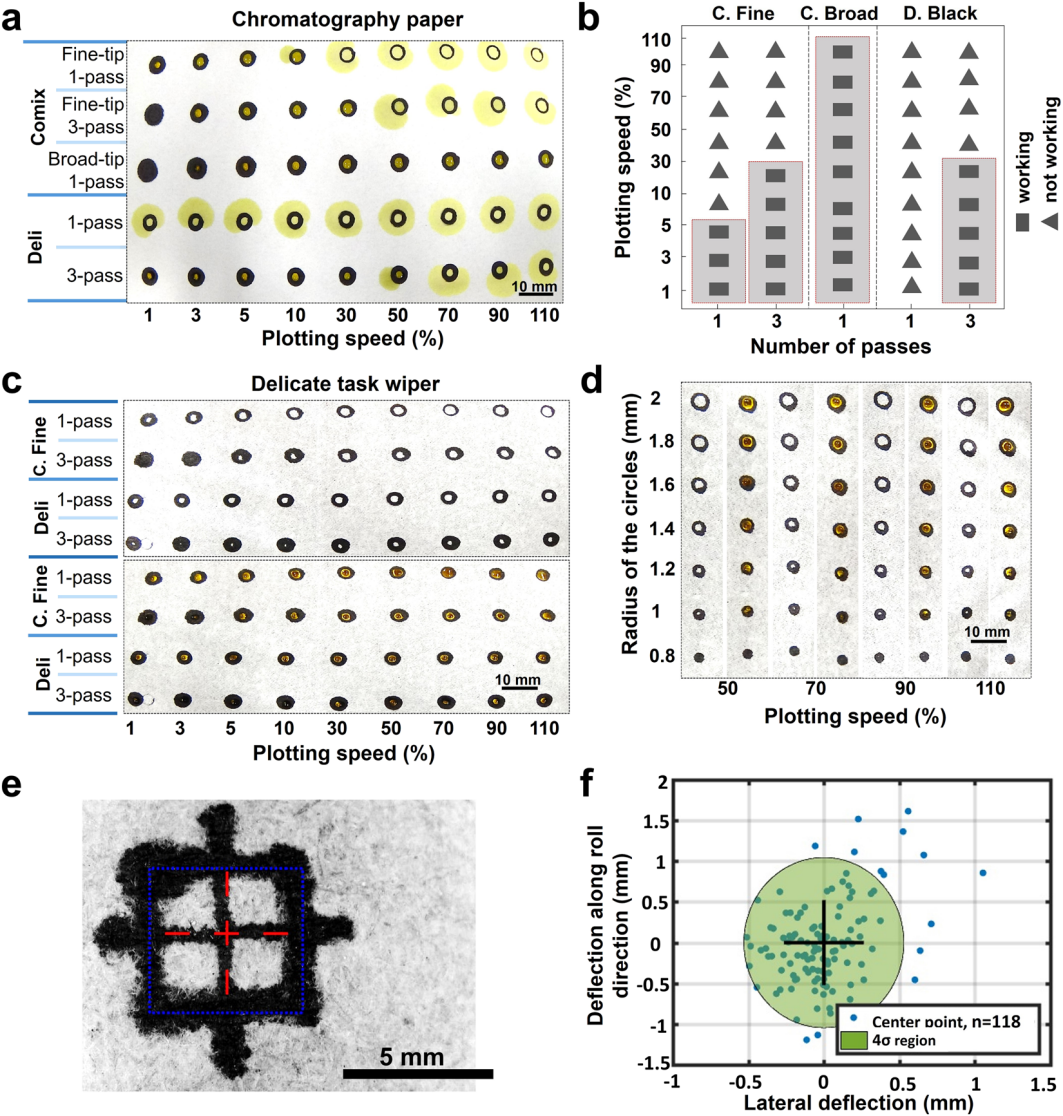
Investigating the effect of plotting speed and pattern dimensions. Testing the water-resistant capability of the patterns by yellow aqueous solution at different plotting speeds, markers, and number of passes on (**a**) Chromatography paper and (**b**) its summarized results as a performance chart. (**c**) Comparing different plotting speeds, markers, and number of passes on delicate task wiper. (**d**) Testing different plotting speeds and pattern dimensions with Comix fine-tip marker on delicate task wipers with a single pass. (**e**) A sample of an image processed pattern used for repeatability assessment. (**f**) Deviation of the plotted pattern from the center of the plotter origin.

Plotting speed is directly related to the amount of ink that diffuses through the paper. Therefore, we can increase the amount of diffused ink and thereby increase the likelihood of achieving water-resistant patterns by decreasing printing speed. To show the penetration of the ink through the paper, we first plotted lines on chromatography paper with different plotting speed and number of passes, and then demonstrated the cross-sections using optical microscope (Fig. 3b). Figure 4a shows that plotting on chromatography paper using Comix fine-tip markers at speeds less than 5% yield water-resistant patterns, and if we increase the number of passes to 3, water-resistant patterns can be achieved at a speed as high as 30%, which is similar to the results for Deli black markers. However, using Comix broad-tip markers for plotting can produce water-resistant patterns at all speeds. Figure 4b summarizes the performance of Deli black and both fine- and broad-tip Comix markers for creating hydrophobic barriers on chromatography paper. The same experiments were performed with Comix fine-tip and Deli black markers on delicate task wipers, which resulted in water-resistant patterns for all tested speeds. These two markers were chosen for testing on delicate task wipers because all other marker types yielded ink-filled patterns. The results of this experiment are presented in Fig. 4c. Moreover, we measured the viscosity and contact angle of the Comix and Deli black inks. The results are presented in Table 1 and Fig. 3c,d,e. Contact angle of the surface covered with Deli ink is less than that of covered by Comix which confirms that Comix ink is more hydrophobic and acts as a better hydrophobic barrier in spite of its lower viscosity.

**Table 1:** Viscosity and contact angle of Comix and Deli ink.

	Comix	Deli
Viscosity (cp) at 23 °C	4.43 ± 0.04	5.94 ± 0.07
Contact angle on chromatography paper	127.6 ± 5.3	9.1 ± 2.6
Contact angle on wiper	119.6 ± 8.8	86.2 ± 5.3

In addition to investigating the effect of plotting speeds, circles with different diameters (from 1.6 mm to 4 mm) were plotted to determine the resolution achievable with AxiDraw while still producing water-resistant patterns. Figure 4d shows the patterns at different sizes and speeds on delicate task wipers plotted with a Comix fine-tip marker. Results showed that the smallest feature which can be plotted without being filled with ink is a circle with a diameter of 2 mm.

### Paper feeder

The result of the paper feeder accuracy test is obtained by plotting the pattern shown in Fig. 4e, and using image analysis to detect the location of the pattern. The results of this experiment are shown as a scatterplot in Fig. 4f. Each data point in this graph shows the deviation of the center of a pattern relative to the average center position for all patterns. The shaded region indicates the data-points with less than 4σ deviation deflection. Data points falling outside of the green circle in this scatterplot indicate misalignment of the paper being fed due to interference from the markers. Specifically, the paper was not adequately clamped to the base; thus allowing the friction between the paper and the moving marker tips to drag the paper, causing shaking and misalignment. Misalignments can be explained by a number of other factors as well, including the surface roughness of paper and the coefficient of friction between the base and paper. The base has a very smooth surface and low coefficient of friction, allowing the paper to be more easily dragged by the marker tip, which has a higher coefficient of friction. It is important to consider the effect that the surface of the base has on the paper.

### Glucose assay

**Figure 5:**
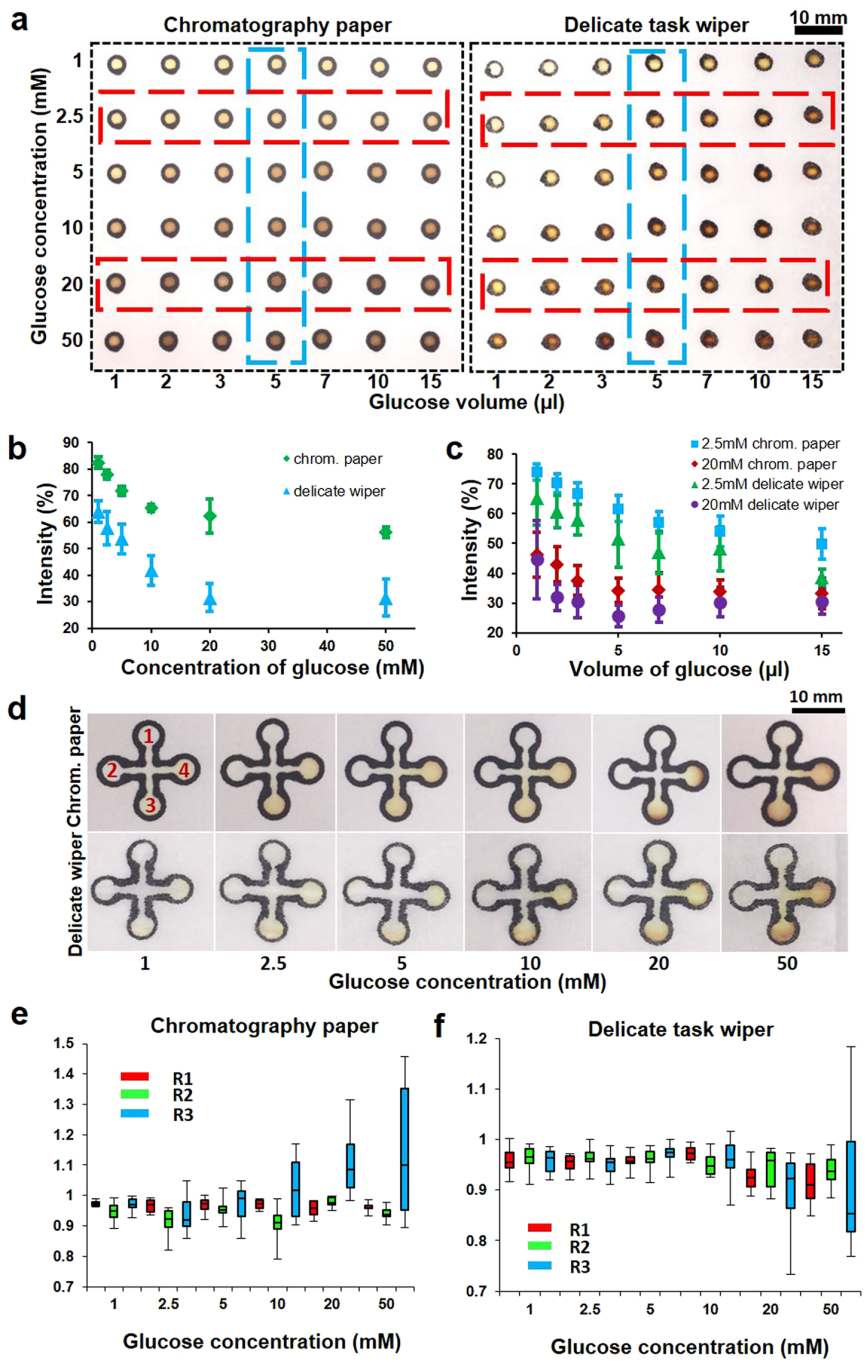
Glucose assay via calorimetric method. (**a**) Effect of glucose concentration and volume on color intensity. (**b**) The quantified results of the effect of glucose concentration on color intensities at constant volumes of 5 µl and 2 µl for chromatography paper and delicate task wipers, respectively. (**c**) The quantified results of the effect of glucose volume on color intensities at concentrations of 2.5 mM and 20 mM on both chromatography paper and delicate task wipers. (**d**) Glucose assay tested at different concentrations on plus-shaped patterns on both chromatography paper and delicate task wipers and (**e**,**f**) their quantified results. Zone 1 is blank, zone 2 has reagent without KI, and zones 3 and 4 have dried reagent. R1 and R2 are the intensity ratios of zone 1 and zone 2 to the paper (*i*.*e*. blank paper color intensity quantified at the outside of plus-shaped patterns using the same image to cancel out the ambient light effects), respectively and R3 is the intensity ratio of zone 3 to zone 4.

To verify the effectiveness of the presented method for fabrication of paper-based microfluidics, a glucose assay was conducted on two different types of paper using colorimetric analysis. Glucose solutions with different concentrations (1, 2.5, 5, 10, 20 and 50 mM) and volumes (1, 2, 3, 5, 7, 10 and 15 µl) were used to show the effect of concentration and volume on color intensity. Figure 5a clearly shows that increasing either concentration or volume of the glucose solution increases color intensity. In the next step, we did the similar experiments at different concentrations of glucose but with a constant volume (2 µl and 5 µl for delicate task wipers and chromatography paper, respectively) and also at different volumes of solution with constant glucose concentrations (2.5 mM and 20 mM). The quantified results of these sets of experiments are presented in Fig. 5b,c, which clearly confirm the direct relationship between color intensity and concentration and volume.

To verify the multiplexing capability of the presented fabrication method in simultaneously running multiple assays on a single device without cross-contamination, we used the plus-shaped patterns with four detection zones. Two of the detection zones were spotted with the glucose reagent and two of them were the designated controls (one with the reagent solution without potassium iodide (KI) and one with no solution). Figure 5d shows that color formation is only observed in the detection zones with the glucose reagent; the control zones were not affected. Figure 5e,f are the quantified results of Fig. 5d, in which R1 and R2 are the ratio of the intensity of detection zone 1 and 2 to paper, respectively and R3 is defined as the intensity ratio of detection zone 3 to 4; since the experimental conditions of the zone 3 and 4 were the same, we expected R3 to be around 1.

## Discussion

Permanent marker inks are usually water-resistant and contain a hydrophobic resin, a solvent (typically ethanol), and a colorant^29^. Patterns were plotted onto chromatography paper and delicate task wipers using permanent markers, and the ink was allowed to penetrate completely through the paper. Chromatography paper was selected because of its uniform thickness and wicking properties while delicate task wipers have a favorably high wicking speed. Based on the commercial information, the thickness of chromatography paper used here is 180 µm, which is consistent with our presented SEM images (Fig. 3a). The thickness of delicate task wipers is not commercially available. Our results showed that thickness of wipers is 55 µm. Based on this information, it can be concluded that the less thickness and larger pore size of the wipers result in better penetration of the ink through the thickness to create barrier.

After evaporation of the solvent, the hydrophobic resin and the colorant remain in the paper and create a hydrophobic barrier throughout the thickness of the paper. Aqueous samples are contained within the hydrophilic zones delimited by the hydrophobic barriers. To assess the water resistance of the permanent markers, aqueous dye was added to circular patterns plotted on both the delicate task wipers and chromatography paper using a desktop pen plotter. As shown in Fig. 2, Comix broad-tip markers on chromatography paper and Comix fine-tip and Deli black markers on delicate task wipers produced sufficiently hydrophobic barriers capable of containing the aqueous dye. However, Deli red and almost all colors of Sipa markers were unable to either effectively resist the aqueous solution on chromatography paper or create unfilled circles with diameter of 4 mm on the delicate task wipers.

Figure 4 shows that Comix broad-tip markers can successfully create hydrophobic patterns at different speeds with a single pass on the chromatography paper, whereas Deli black marker can resist the aqueous sample only at speeds lower than 30% and with 3 passes of printing; this disparity may be due to the difference in hydrophobicity of the marker inks resulting from the variation in composition. The fine-tip of Comix marker produced patterns on the chromatography paper successfully resisted aqueous solution at speeds lower than 5% with a single pass and speeds lower than 30% with 3 passes. The fine-tip Comix marker only works at low speeds because it deposits an insufficient amount of ink that is unable to diffuse entirely through the paper. Moreover, based on the results in Fig. 4, we can use Comix fine-tip markers to have features as small as 2-mm-diameter circles. By considering the overall performance of the markers, fine- and broad-tip Comix markers were selected to continue the experiments to demonstrate high-throughput microfluidics fabrication with an Axidraw plotter.

To show an application of the presented fabrication method in bio-chemical analysis field, we conducted glucose assay. Results (Fig. 5) demonstrate that our device is capable of showing a direct relation between the glucose amount (e.g. by changing both concentration and volume) and resulting color intensity. Figure 5e,f shows the multiplexing ability of the presented method for running simultaneous assays. However, it should be noted that the volume of the spotted sample plays an important role and it should be optimized depending on the type of the paper for preventing the cross-contamination issue.

## Conclusion

Choosing an appropriate patterning method for mass fabrication of paper-based microfluidics requires a trade-off between cost, convenience, and resolution. We have demonstrated an inexpensive, high-throughput, simple, and rapid prototyping method for fabrication of paper-based microfluidics by using a desktop pen plotter, a customized paper feeder, and a permanent marker on delicate task wipers and chromatography paper. With the proposed technique, the fabrication cost of a 10 cm by 100 cm paper microfluidic strips is less than $1 and takes less than 15 min. To demonstrate the potential applications of this fabrication method in biological and chemical analysis, a glucose assay was performed, and the results were quantified. The ink capacity of the commercial permanent marker may be considered as a drawback of this technique regarding mass production, but that could easily be addressed by supplying the marker with a larger ink reservoir. Furthermore, an empty marker could be used for dispensing bioreagents onto the detection zones of paper microfluidics. By considering the cost and resolution offered by this fabrication method as well as ease of fabrication, this approach is highly-suitable for fabrication of paper-based microfluidics for different applications including point-of-care diagnostics, food safety monitoring, and environmental testing, especially in poor-resource areas.

## Materials

A desktop pen plotter, AxiDraw (Evil Mad Scientist Laboratories, CA, US); Deli markers with different colors (Deli group, Zhejiang, China); Sipa markers with different colors (Sino Path Enterprises Ltd., Guangong, China); Double-ended Comix markers (Comix group Co. Ltd., Shenzhen, China), where the fine-tip and broad-tip markers are 0.5 and 2.0 mm in diameter, respectively; delicate task wipers, KIMTECH (Kimberly-Clark Worldwide, Inc. GA, US); chromatography paper, Whatman No. 1 (GE healthcare life sciences, IL, US); glucose oxidase/peroxidase reagent (G 3660), potassium iodide (793582), trehalose (PHR1344) all from Sigma- Aldrich, MO, US; pure glucose (Modernist Pantry LLC., NH, US); a microcontroller, Arduino nano (Arduino LLC, US).

## Methods

### The effects of marker and paper

To investigate the capability of the markers to create hydrophobic barriers resisting aqueous solution, we tested various commercial brands of markers including Sipa (oil-basedmarker), Deli and Comix (permanent markers). The patterns, circles with a diameter of 4 mm, were prepared withSolidWorks and plotted by AxiDraw pen plotter on two different types of paper (chromatography paper and delicatetask wipers). The water-resistant performance of the patterns plotted by six different colors of Sipa markers,two different colors of Deli markers, and one color of double-ended Comix markers (both fine- and broad-tips)were investigated by spotting aqueous solution of yellow food dye in the center of the patterns. To increase theamount of ink diffusing through the paper, multiple passes of plotting were performed.

### The effect of plotting speed and pattern dimensions

AxiDraw is a desktop pen plotter with XY resolution of 80 steps per mm, with ±0.1 mm reproducibility, and a maximum plotting speed of 177 mm/s. The speed is adjustable and directly affects the amount of ink diffused through the paper and the feature size of the plotted patterns. A range of speeds from minimum (1%) to maximum (110%) was tested for plotting circles with diameters from 1.6 to 4 mm. Comix (both fine- and broad-tip) and black Deli markers were tested for this set of experiments as they successfully created hydrophobic barriers.

### Paper feeder

To achieve a high-throughput method for fabrication of paper-based microfluidics, we developed a paper feeding mechanism synchronized with AxiDraw desktop pen plotter. We created the custom paper feeder using a feeder and its motor from an inkjet printer (HP OfficeJet 6500 A). The feeder consists of two rollers (1.6 cm in diameter) with a rubber coating to grip the paper. The rollers push the paper down onto the base to keep it inline and level during patterning. An electrical circuit consisting of an external DC power supply, a Mosfet, and a microcontroller is used to control the entire system through a PC. Using an open-loop controlling algorithm, the microcontroller controls the rotation of the motor in accordance with the pattern size and drawing speed. To activate the roller, the electrical circuit is powered by 5 volts and 0.5 amperes, provided by the external DC power supply. The roll of paper is held using a bracket (Fig. 1c) designed with SolidWorks 2014 (Dassault Systèmes SolidWorks Corp., France) and cut from a 3 mm-thick acrylic plate with a CO2 Laser Cutter (Universal Laser Systems, Inc., AZ, US). The holder and feeder are placed before and after the pen plotter, respectively (Fig. 1d).

The accuracy of the paper feeder was tested to assess its performance. The microcontroller was programmed to run the motor for 1 sec to unravel 10 mm of the paper roll, then stop for 10 sec to allow Axidraw to plot a 5 by 5 mm square. All squares were plotted at the same XY position by sending the plot command via the PC. A Carson eFlex digital camera (Carson Optical, Inc., NY, US) was used to capture images at a fixed position, focused on the center of the square, for analyzing the position of patterns. 120 repetitions were performed, and the images were analyzed using a MATLAB script (MathWorks, MA, US) to measure the repeatability and accuracy of the feeder.

### Glucose assay

To demonstrate the applicability of the proposed fabrication method for paper-based microfluidics in bioassays and verify their effectiveness, we conducted a glucose assay with different concentrations and volumes of glucose solutions (1–50 mM and 1–15 μl). This method relies on the color shift from clear to brown due to the enzymatic oxidation of iodide (I−) to iodine (I2). First, glucose is oxidized to produce hydrogen peroxidase (Equation (1)), which can enzymatically oxidize iodide (due to the presence of KI in reagent) to iodine in the presence of horse radish peroxidase (HRP) and produce water as a by product (Equation (2))^12, 15, 30–32^.

1 $${Glucose}+{H}_{2}O+{O}_{2}\,\mathop{\longrightarrow }\limits^{G{O}_{x}}\,Gluconic\,Acid+{H}_{2}{O}_{2}$$

2 $$Chromogen+{H}_{2}{O}_{2}\,\mathop{\longrightarrow }\limits^{peroxidase}\,Dye+{H}_{2}O$$

Using the fine-tip Comix marker, circular patterns with a diameter of 4 mm and plus-shaped patterns with 4 detection zones and 4 channels were plotted on both delicate task wipers and chromatography paper with speeds of 80% and 4%, respectively (Fig. 4a,d). To show the effect of glucose concentration and solution volume on the resulting color, we tested a range of concentrations (1–50 mM) and volumes (1–15 μl) on circular patterns. Additionally, we spotted 7 μl and 35 μl of glucose solution with different concentrations onto the center of the plus-shaped patterns plotted on wiper and chromatography paper, respectively. To activate the detection zones, 0.5 μl and 1 μl of the reagent solution (for wipers and chromatography paper, respectively) were spotted in each detection zone and left to dry at room temperature for 10 minutes. The reagent solution includes glucose oxidase/ peroxidase reagent (125 units of glucose oxidase enzyme activity and 25 units of peroxidase enzyme activity per ml of solution), 0.3 M trehalose and 0.6 M potassium iodide^15^. Trehalose was added to the reagent mixture as a stabilizer for the proteins in their active form. It has been shown that an absence trehalose yields a loss of enzymatic activity^32^.
